# Integrating Experimentation and Quantitative Modeling to Enhance Discovery of Beta Amyloid Lowering Therapeutics for Alzheimer’s Disease

**DOI:** 10.3389/fphar.2012.00177

**Published:** 2012-10-04

**Authors:** Yasong Lu

**Affiliations:** ^1^Department of Pharmacokinetics, Dynamics and Metabolism, Pfizer Worldwide Research and DevelopmentGroton, CT, USA

**Keywords:** Aβ, experimentation, quantitative modeling, efficiency, drug discovery

## Abstract

Drug discovery can benefit from a proactive-knowledge-attainment philosophy which strategically integrates experimentation and pharmacokinetic/pharmacodynamic (PK/PD) modeling. Our programs for Alzheimer’s disease (AD) illustrate such an approach. Compounds that inhibit the generation of brain beta amyloid (Aβ), especially Aβ42, are being pursued as potential disease-modifying therapeutics. Complexities in the PK/Aβ relationship for these compounds have been observed and the data require an advanced approach for analysis. We established a semimechanistic PK/PD model that can describe the PK/Aβ data by accounting for Aβ generation and clearance. The modeling characterizes the *in vivo* PD (i.e., Aβ lowering) properties of compounds and generates insights about the salient biological systems. The learning from the modeling enables us to establish a framework for predicting *in vivo* Aβ lowering from *in vitro* parameters.

## Introduction

The pharmaceutical industry has been experiencing a decrease in productivity despite increasing R&D investment (FDA, 2004; Bunnage, 2011). Approaches for improving performance have been proposed from organizational (Sams-Dodd, 2005), operational (Bunnage, 2011; Johnstone et al., 2011; Knutsen, 2011; Elebring et al., 2012), and scientific perspectives (FDA, 2004; EMEA, 2007; Zhang et al., 2008; Morgan et al., 2012). Some researchers have argued that an increase in productivity may arise from a revamp of early discovery (Dimitri, 2011; Knutsen, 2011). Choosing the appropriate targets and optimal compounds in discovery should increase the probability of success at later stages (Bunnage, 2011; Maurer, 2011).

In the neuroscience area, Alzheimer’s disease (AD) and dementia represent an urgent and significant unmet medical need. Drugs which temporarily alleviate some symptoms are on the market, but disease-modifying drugs which slow AD progression remain unavailable. While there are still uncertainties about AD etiology, the current leading hypothesis, known as amyloid cascade hypothesis (Hardy and Higgins, 1992), posits that AD is caused by an abnormal accumulation in the brain of amyloid beta (Aβ), a protein with a molecular weight of ∼4 kDa. Aβ is generated through sequential enzymatic cleavages of amyloid precursor protein, first by beta secretase (BACE1) and then by gamma secretase (GSI; reviewed in De Strooper et al., 2010). Aβ has numerous forms depending on the exact cleavage site which dictates the length of the resulting amino acid sequence. Aβ40 and Aβ42 are the main forms found in the amyloid plaques in Alzheimer’s brains (Gravina et al., 1995), and Aβ42 is the predominant toxic form (El-Agnaf et al., 2000). Reduction of Aβ, particularly Aβ42, in the brain therefore has been proposed as a potential disease-modifying treatment for AD. Potential therapies include small molecules that inhibit BACE1 (BACEi) or gamma secretase (GSI) to lower total Aβ production, or that modulate gamma secretase (GSM) to lower Aβ42 selectively. All three approaches are being pursued in the pharmaceutical industry (reviewed in Ghosh et al., 2008; Imbimbo, 2008; Pettersson et al., 2011).

BACEi, GSI, or GSM programs seek compounds to test rigorously and definitely the amyloid cascade hypothesis in the clinic. Such compounds should be capable of distributing to the target site, interacting with the target, and eliciting sufficient pharmacodynamic (PD) response, i.e., Aβ lowering, in humans at concentrations that afford an acceptable safety margin. Preclinical identification of such compounds is based on intensive evaluation of pharmacokinetics (PK), PD, and safety in *in vitro* assays and preclinical animal models. This identification process is more efficient when the *in vivo* pharmacology and relevant biological systems are well understood.

A relevant *in vivo* measure of modulating secretase activities is brain Aβ42 lowering, which in practice can be assessed only in preclinical species, typically rodents. In addition, CSF Aβ40 and Aβ42 are often monitored for their potential use as biomarkers for brain Aβ lowering. Numerous data sets from in-house and external studies have demonstrated complexities in the PK/PD relationship for Aβ lowering agents which pose challenges for both characterizing compounds’ *in vivo* PD properties and translating effects across species. We have established a semimechanistically based PK/PD model to analyze PK/Aβ data, and through its application have obtained reasonable characterization of compounds’ *in vivo* PD properties and Aβ clearance kinetics (Wang et al., 2010; Lu et al., 2011, 2012a,b,c). Here, I summarize our systematic learning from quantitative modeling of the Aβ data, and advocate for the integration of experimentation and PK/PD modeling using the BACEi, GSI, and GSM projects as an example. In this article, PD therefore refers to Aβ lowering in the brain or CSF. Whether or not lowering brain Aβ in patients will translate to clinical benefits is beyond the scope of this article.

## Complexities in PK/Aβ Data

**Figure 1:**
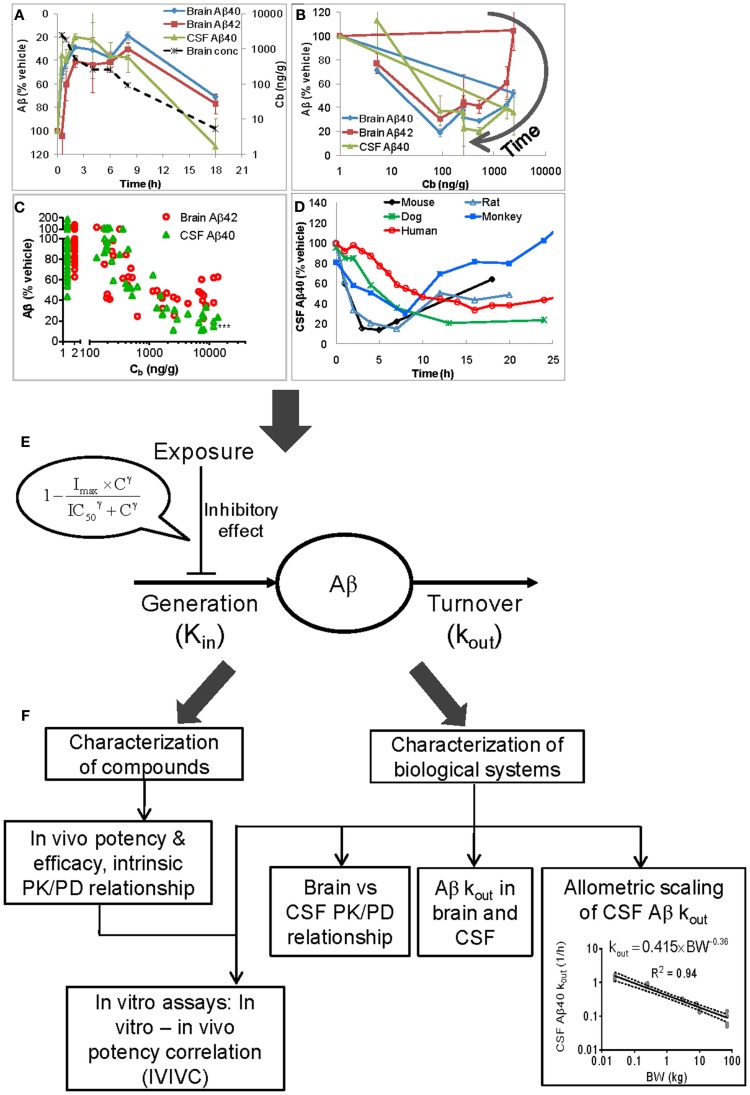
**Inherent complexities in PK/Aβ data (A–D), the semimechanistically based PK/PD model (E) for analyzing Aβ data, and the insights from the modeling (F)**. The complexities in the data are reflected by hysteresis **(A,B)**, differences in the effect size between brain and CSF **(A,C)** and in the temporal profiles for brain and CSF Aβ **(A)**, and variation of CSF Aβ temporal profile across species **(D)**. **(A)**: Time course data from 129/SVE mice treated orally with LY450139 (GSI) at 150 mg/kg (Lu et al., 2011). The effect time courses lag temporally behind the brain concentration time course. **(B)** The time course data in **(A)** plotted as Aβ vs. exposure to illustrate the hysteresis loops, where the effect does not correlate strictly with drug concentration, and instead also depends on time. **(A,B)** suggest a delay between drug concentration and manifestation of an effect, a phenomenon known as hysteresis. **(C)** Data at 3 h post-dosing from 129/SVE mice treated subcutaneously with LY2811376 (BACEi) at 1, 3, 10, 30, and 100 mg/kg (reproduced from Lu et al., 2012c with permission from American Society for Pharmacology and Experimental Therapeutics). Each symbol represents an individual animal. The individuals in the 1–2 ng/g range are vehicle controls, adjusted from the actual concentration of zero for illustration on the logarithmic scale. **(D)** The mean time course profiles of CSF Aβ40 in the 129/SVE mouse, Sprague-Dawley rat, cynomolgus monkey, beagle dog, and healthy human subject treated with LY2811376 at 100 mg/kg, subcutaneously, 50 mg/kg, orally, 20 mg/kg, orally, 5 mg/kg, orally, and 90 mg, orally, respectively. **(E)** The semimechanistic model assumes that the Aβ level is controlled by a zero-order generation rate, which is modified by an inhibitory effect due to BACEi, GSI, or GSM, and a first-order clearance process. **(F)** The modeling enables characterization of compounds’ *in vivo* PD properties and the relevant biological systems. The plot of allometric scaling of CSF Aβ40 *k*_out_ is reproduced from (Lu et al., 2012a) with permission from S. Karger AG, Basel, Switzerland, and updated with inclusion of the rat. By reversing the directions of all arrows, this figure illustrates an integrative framework for projecting compounds’ *in vivo* PD behaviors from *in vitro* and system parameters. Hysteresis: A tendency for an effect profile to lag temporally behind an exposure profile after drug treatment. Plotting Aβ levels vs. the concurrent exposures yields a hysteresis loop; the effect does not correlate strictly with concentration, and instead also depends on time. Hysteresis demonstrates an apparent lack of exposure-response relationship. Analysis of PK/PD data with hysteresis requires complex models, such as a link model, an indirect response model, or a mechanistically based model (Mager et al., 2003; Danhof et al., 2007). Cb, drug brain concentration; *K*_in_, Aβ generation rate; *k*_out_, first-order rate constant for Aβ clearance; *I*_max_, maximum inhibition of *K*_in_; IC_50_, concentration that causes half-maximum inhibition of *K*_in_; γ, Hill coefficient; BW, body weight.

The relationship between the PK and Aβ data for BACEi, GSI, and GSM is complex. First, Aβ lowering after compound treatment shows hysteresis (Figures 1A,B; Hawkins et al., 2011; Lu et al., 2011, 2012c), a tendency for an effect profile to lag temporally behind an exposure profile. Plotting Aβ levels vs. the concurrent exposures yields a hysteresis loop; the effect does not correlate strictly with concentration, and instead also depends on time (as can be seen in Figure 1B). Second, within a given species (mouse, rat, or guinea pig), the data from single-time-point sampling often show stronger Aβ lowering in CSF than in brain with the discrepancy widening as the dose increases (Figure 1C; Wang et al., 2010; Lu et al., 2012c). Third, following dosing the time courses of CSF and brain Aβ diverge from one another. Figure 1A illustrates this behavior observed in the mouse, rat, and guinea pig; CSF Aβ decreases and returns to baseline more rapidly than brain Aβ (Lu et al., 2011, 2012c). The separation is increasingly pronounced with dose (Wang et al., 2010). Fourth, the shape of the CSF Aβ profile varies across species. The shape becomes more blunted with increasing body size (Figure 1D). These observations provoke a series of critical questions:

- How should we appropriately characterize a compound’s *in vivo* PD properties (potency and efficacy)?
- Why are there differences in the effect size and temporal profile between brain and CSF and across species?
- Is CSF Aβ a valid biomarker for brain Aβ lowering given the discrepancy in Aβ lowering between the two compartments?
- Are the mouse and rat suitable pharmacology models for humans, and if yes, how should we scale an Aβ lowering effect from these species to humans?

Each question represents a substantial hurdle for rational and efficient discovery. It is therefore critical to seek a sound mechanistic understanding of the complexities and obtain answers to these questions.

## A Semimechanistically Based PK/PD Model for Analyzing Aβ Data

The hysteresis precludes the use of the classical sigmoidal model which assumes that the PD results from the concurrent drug concentration. A more sophisticated model is thus necessary. We established a semimechanistic model that can describe the complex PK/Aβ data by taking Aβ generation and clearance into consideration (Lu et al., 2011, 2012a,c). As shown in Figure 1E, this model assumes that the level of steady-state Aβ in a compartment is maintained via the balancing of a zero-order generation rate (K_in_) and a first-order clearance process (with a fractional turnover rate of *k*_out_). Consistent with the mode of action of BACEi, GSI, or GSM, an inhibitory effect is described by a sigmoidal term that modifies the generation rate K_in_ (Eq. 1). The drug concentration (C) may be that in plasma, brain, or CSF depending upon the data available. This model represents a biologically reasonable simplification of Aβ homeostasis and its pharmacological modulation.

(1) $$\frac{dA\beta }{dt}=K_{\text{in}}\times \left(1-\frac{I_{\max}\times C^{\gamma }}{I{C_{50}}^{\gamma }+C^{\gamma }}\right)-k_{\text{out}}\times A\beta$$

To remove potential confounding non-specific effects, the absolute Aβ levels in treatment groups are expressed as percentages of the concurrent vehicle control and the normalized time courses are then modeled. By fitting the normalized data, the model produces estimates for the unknown parameters: *k*_out_, *I*_max_ (maximum inhibition of *K*_in_), IC_50_ (concentration at which 50% of *I*_max_ is achieved), and γ (Hill coefficient). At steady-state, Eq. 1 simplifies to *K*_in_ = 100% × *k*_out_ since in this scenario the Aβ level is constant at 100% of the basal level. Once *k*_out_ is estimated, *K*_in_ can be readily calculated.

## Insights from PK/PD Modeling

The PK/PD modeling extracts, from complex PK/Aβ data, parameters that allow appropriate characterization of the properties of compounds’ *in vivo* pharmacology and the pertinent biological systems.

### Characterization of a compound’s *in vivo* PD properties

A compound’s *in vivo* potency and efficacy for lowering Aβ can be defined by the modeling-derived IC_50_ and *I*_max_, respectively. For estimation of steady-state average Aβ lowering after treatment, a relationship between exposure and the modified Aβ generation rate, *R*_gen_ as a fraction of the control is defined by Eq. 2

(2) $$R_{\text{gen}}=\frac{K_{\text{in}}\times \left(1-\frac{I_{\max}\times C^{\gamma }}{I{C_{50}}^{\gamma }+C^{\gamma }}\right)}{K_{\text{in}}}=1-\frac{I_{\max}\times C^{\gamma }}{I{C_{50}}^{\gamma }+C^{\gamma }}$$

This exposure – *R*_gen_ relationship defines the intrinsic PK/PD relationship for a BACEi, GSI, or GSM. It is devoid of confounds arising from PK behaviors or Aβ turnover kinetics. Mathematically, this intrinsic PK/PD relationship is equivalent to the relationship between an exposure and time-weighted-average Aβ lowering at steady-state after repeated dosing.

Empirical non-modeling based analyses of PK/Aβ data may involve several approaches, such as single-time-point exposure/Aβ assessment, area-under-the-concentration-curve (AUC) vs. maximum Aβ lowering, or AUC vs. area-under-the-Aβ-curve assessment. As discussed earlier (Lu et al., 2011), these approaches have serious flaws and limitations. They not only lack necessary predictive or extrapolating power, but also likely yield misleading or erroneous potency estimates for a compound. Therefore, for the discovery of Aβ lowering therapeutics, these approaches should be replaced by quantitative PK/PD modeling.

### Characterization of pertinent biological systems

The biological systems involved in the discovery of Aβ lowering therapeutics include *in vitro* assays for high throughput screening and animal models such as mice, rats, guinea pigs, dogs, monkeys, or humans for *in vivo* pharmacology evaluation.

#### *In vitro* assays

An *in vitro* assay provides a measure of potency (IC_50_), which is one of the primary *in vitro* parameters for directing synthesis of new compounds and for triaging, rank ordering, and prioritizing among existing compounds. Before an *in vitro* assay can be relied upon, a validation from the translational pharmacology standpoint has to be conducted to assess how the *in vitro* potency is correlated with *in vivo* potency. With *in vivo* potency estimated by PK/PD modeling, an *in vitro* – *in vivo* potency correlation (IVIVC) may be established. The IVIVC will allow selection of the most relevant *in vitro* assay and determination of the quantitative *in vitro* – *in vivo* translation.

#### Animal models

Through PK/PD modeling of extensive data sets, we have determined the turnover rate (*k*_out_) for Aβ in the brain and CSF in the mouse (non-transgenic), rat, and guinea pig. The *k*_out_ in brain is slower than in CSF in all three species. In the mouse, the *k*_out_ for brain Aβ42 is approximately threefold lower than that for CSF Aβ40 (0.49 vs. 1.42/h; Lu et al., 2012a), and in the rat and guinea pig, the *k*_out_ for brain Aβ42 is about twofold lower than that for CSF Aβ42 (Lu et al., 2012c). The difference in the *k*_out_ leads to distinct Aβ profiles and effect sizes at a given sampling point in the two compartments following treatment (Lu et al., 2012a).

We have also determined the *k*_out_ for CSF Aβ40 in the rat, dog, monkey, and human in addition to the mouse. The *k*_out_ value scales allometrically across these five species, following the equation *k*_out_ = 0.415 × BW^−0.36^, where BW represents the body weight (Figure 1F). The decrease in the *k*_out_ with body weight causes the increasingly blunted shapes of the CSF Aβ40 time course (Lu et al., 2012a).

CSF Aβ is thought to be primarily derived from the brain. As long as the PK/PD relationship for Aβ lowering in CSF is consistently related to that in brain, CSF Aβ may serve as a biomarker for brain Aβ lowering despite the discrepancy in Aβ profiles in the two compartments. Our modeling analyses of seven compounds, across the three mechanisms (BACEi, GSI, and GSM), in three species (mouse, rat, and guinea pig) demonstrated a consistent overlap of the intrinsic PK/PD relationships for brain and CSF over the range of 0–50% lowering in *R*_gen_ (Lu et al., 2012c). This analysis supports CSF Aβ as a potential biomarker for brain Aβ lowering from the clinical trial standpoint.

Another conundrum that modeling can shed light on is which preclinical species are suitable pharmacology models for evaluating BACEi, GSI, and GSM. The choice is generally a compromise between multiple factors, such as animal cost (direct cost, husbandry, and genotyping if applicable), animal size (relevant to chemical scale up), homology of Aβ sequence (human shares the same sequence with the guinea pig, dog, and monkey, but not mice or rats), streamlined operation (preferentially the same species for pharmacology evaluation, absorption, distribution, metabolism, and elimination profiling, and safety characterization), and predictivity for human Aβ lowering. The predictivity for human Aβ lowering can be assessed based on interspecies comparisons of the intrinsic PK/PD relationship. When the interspecies translation is understood, a rational choice of the suitable model may be made. In this regard, two examples have been reported. (1) The transgenic Tg2576 mouse (Hsiao et al., 1996) has been recognized as a model for AD, and traditionally has been used to identify GSI despite the tremendous cost. Since the primary PD endpoint for GSI is Aβ lowering in the central compartments, a non-transgenic strain may be suitable if the PK/PD relationship in this strain is predictive of that in humans. Therefore, we undertook comprehensive PK/PD analyses for several GSI compounds, including LY450139 and BMS-708163, in the Tg2576 mouse, non-transgenic 129/SVE mouse, rat, guinea pig, dog, monkey, and human. Our analyses indicated that the intrinsic PK/PD relationship for brain Aβ42 and CSF Aβ40 in the 129/SVE mouse is consistent with, and hence predictive of, that observed for CSF Aβ in humans (Lu et al., 2011). As a result, we suggested the 129/SVE mouse as a reasonable model for the discovery of GSI (Lu et al., 2011), allowing for a drastic reduction in the cost on animals. (2) For identification of GSM, the guinea pig was thought to be a preferred model due to its Aβ sequence being identical to that of human. This choice was not ideal as guinea pigs are generally not used for PK or safety profiling. To address the conflict, we collected PK/Aβ data for two structurally distinct GSM in the two species. Our modeling analyses demonstrated a similar intrinsic PK/PD relationship for Aβ42 lowering in the central compartments in guinea pig and rat (Lu et al., 2012b). Although the predictivity of these species for humans remains unknown due to lack of available clinical data, this result suggests that the difference in the Aβ sequence has no bearing on the PD response and thus justifies the substitution of rats for guinea pigs for GSM evaluation.

## Uses of PK/PD Modeling and Simulation

**Figure 2:**
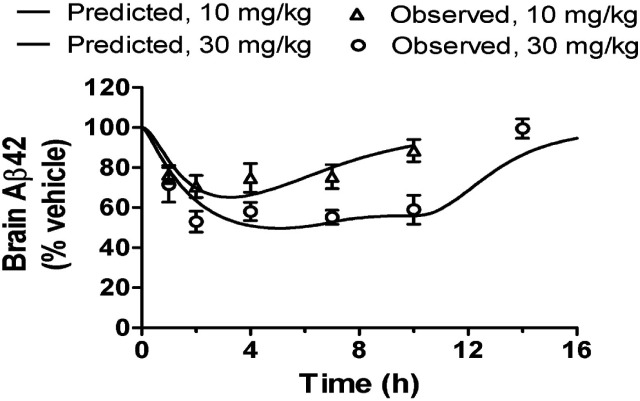
**The time courses of brain Aβ42 in the mouse after an oral dose of a BACEi are predicted adequately using the PK/PD model with the *I*_max_, IC_50_, γ, and *k*_out_ determined from a separate study with subcutaneous administration of the same compound**. This example demonstrates the predictivity of the PK/PD model suitable for enhancing the discovery research.

An Aβ time course profile is determined by three components, exposure time course, intrinsic PK/PD relationship, and Aβ turnover rate (Lu et al., 2011). Once understood, these components may be re-integrated using a PK/PD model to predict the time course of Aβ concentrations after a treatment (Lu et al., 2012a,c). This exercise has several advantages for discovery research. First, it may replace studies that do not necessarily generate new information. For example, if a compound is characterized for its PK/PD relationship for Aβ lowering at 5 and 30 mg/kg subcutaneously, one may later be interested in its effects at 10 mg/kg orally. With an oral PK time course at 10 mg/kg, along with the intrinsic PK/PD relationship and Aβ turnover rate derived earlier, a PD profile can be predicted from simulations (see an example in Figure 2; the data set not used in the model development is predicted adequately by the model.) As such, the oral PD time course study is obviated. Second, it can guide design of preclinical studies and clinical trials. The predictions of Aβ time courses facilitate selections of appropriate doses and sampling time points to ensure the outcomes are informative. We have been using this approach routinely to design our studies in multiple preclinical species. The high quality of the data from such studies has been appreciated internally. Third, modeling and simulation is a useful tool for hypothesis testing. While preclinical PK/PD characterization is often conducted in an acute, single-dose setting, a chronic, repeated-dosing regimen is generally clinically relevant. It is therefore necessary to assess whether the PK/PD relationship shifts after repeated dosing. With the null hypothesis of no shift, the simulations of Aβ profiles after chronic treatment are then compared with observations from a subsequent chronic preclinical study. The agreement or disagreement between the two allows inference of failure to reject the null hypothesis or not. These applications of the modeling and simulation ensure a return of informative data and knowledge on a given resource investment.

## Sufficient Experimental Data to Enable Parameter Estimation

The utility of PK/PD modeling and simulation is contingent on reliable estimation of the unknown parameters, *k*_out_, *I*_max_, IC_50_, and γ, which, in turn, is driven by sufficiently informative data. Note that “sufficiently informative data” is fundamentally different from “lots of data;” rather, it is characterized by wide coverage of the dynamic range (from minimal to near maximum lowering of Aβ) and ample time course sampling points (capturing the effect onset and offset phases). Single-time-point dose – response studies, commonly conducted for the BACEi, GSI, and GSM programs in the industry, carry less information because they only capture a snapshot of whole time courses. In our practice, we have de-prioritized dose – response studies and directed resources to well-designed time course studies (Wang et al., 2010; Lu et al., 2011, 2012a,b,c). While time course studies appear to require more resources, we believe that the value that these studies bring warrants the investment.

## Reactive Data Collection vs. Proactive-Knowledge-Attainment

It is common that a discovery process is driven by identification of key compounds. That is, once meeting certain criteria, a compound is fully profiled for PK and PD properties. If a detrimental liability is later identified, the compound, along with all the relevant data, is then abandoned. As soon as another compound hits the criteria, the next cycle starts. In this process, data are collected reactive to the identification of presumably viable compounds, and little systematic knowledge is accumulated along the way. The insights from and uses of modeling discussed above stem from the proactive-knowledge-attainment philosophy implemented in our BACEi, GSI, and GSM programs. With this philosophy, a discovery program can be executed in two phases: accumulating necessary systematic knowledge using compounds of diverse properties irrespective of their druggability, and applying the knowledge to guide lead optimization, compound selection, and pharmacological translation to the clinic. This strategy is likely to enhance the efficiency of discovery research, and is of similar spirit to the proposals by other researchers (Mager et al., 2009; Maurer, 2011; van der Graaf and Benson, 2011).

## Summary

Small molecule BACEi, GSI, and GSM have been pursued as potential disease-modifying drugs for AD. The preclinical pharmacological activities of these compounds are assessed primarily with the reduction in brain and CSF Aβ. Complexities in the PK/Aβ data for these compounds have been observed, and require improved approaches for analysis. Using a semimechanistically based PK/PD model, we are able to characterize reasonably the *in vivo* PD properties of compounds and the relevant biological systems. This characterization enables establishment of an integrative framework for predicting a compound’s *in vivo* PD behaviors from *in vitro* parameters. The proactive-knowledge-attainment philosophy has driven the research operations of our BACEi, GSI, and GSM programs. With intensive experimentation and modeling analyses, we have achieved a plausible mechanistic understanding of the apparent discrepancy of Aβ lowering profiles across compartments and across species, and have been able to choose suitable species, e.g., non-transgenic over Tg2576 mouse, rat over guinea pig, for preclinical *in vivo* assessment. Given the values it has brought, this proactive-knowledge-attainment philosophy is expected to enhance our ability to select high quality compounds for clinical testing.

## Conflict of Interest Statement

Yasong Lu was an employee of Pfizer Inc. when preparing this article.
